# Transcript levels of phytoene desaturase gene in *Dunaliella salina* Teod. as affected by PbS nanoparticles and light intensity

**Published:** 2016-09

**Authors:** Hajar Zamani, Ali Moradshahi

**Affiliations:** Department of Biology, College of Sciences, Shiraz University, Shiraz, Iran

**Keywords:** Phytoene desaturase, qRT-PCR, Carotenoids, Lead sulfide

## Abstract

Phytoene synthase (Psy) and Phytoene desaturase (Pds) are the first two regulatory enzymes in the carotenoids biosynthetic pathway. The genes *Psy *and *Pds *are under transcriptional control in many photosynthetic organisms. In the present study, using quantitative real time- PCR (qRT-PCR), the effects of uncoated and gum-Arabic coated PbS nanoparticles (GA-coated PbS NPs) and light intensity on the mRNA levels of *Pds *were investigated. Relative to mRNA level of *Pds *at 100 µmol photon m-2 s-1 light intensity (control culture), 2.2-fold increase in transcript levels occurred after 12 h of exposure to higher light intensity, which is significantly (P<0.05) different compared to control. After 48 h of exposure, the mRNA level of *Pds *was reduced to that in control. This indicates that light intensity regulates *Pds *at the mRNA level. In the presence of uncoated and GA-coated PbS NPs, the transcript levels of *Pds *were decreased over time, with uncoated PbS NPs having more inhibitory effects on mRNA levels compared to GA- coated PbS NPs. This shows that PbS NPs have adverse effects on transcription or post transcriptional processing and coating nanoparticles with biopolymers reduces their toxicity to organisms. Being under control, it seems that genetic manipulation of *Pds *may result in increased biotechnological production of carotenoids by *D. salina*.

## INTRODUCTION

*Dunaliella salina *Teod. is a unicellular green algae that accumulates large quantities of β-carotene under certain environmental condition such as nutrient limitation, high salinity, high light intensity and temperature stress [1]. It has been suggested that β- carotene overproduction by high light intensity requires activation of gene(s) encoding β- carotene biosynthetic enzyme(s) [2]. In *Dunaliella, *carotenoids are mainly synthesized in the plastids via the methylerythritol phosphate (MEP) pathway [3, 4]. In this pathway, phytoene synthase (Psy) and phytoene desaturase (Pds) seem to be under transcriptional control and have important role in the regulation of carotenoids biosynthesis [5, 6]. Psy catalyzes the head to tail condensation of two molecules of 20-C compound geranylgeranyl pyrophosphate (GGPP) to produce the 40-C compound phytoene which is desaturated to ζ-carotene by Pds. Through several enzymatic steps ζ-carotene is converted to lycopene and then to β- carotene [7].

There are controversial reports on the protein levels and mRNA of *Psy *and *Pds *in *D. salina *under environmental stresses, such as high light intensity and nutrient limitation. Sanchez-Estudillo et al. [8], reported no change in the steady-state mRNA levels of *Psy *in *D. salina *grown under nitrogen deficiency. In contrast, several fold increase in mRNA levels of *Psy *and *Pds *was observed under nutrient limitation by Coesel et al. [9]. Possible changes in the mRNA levels of *Psy *and *Pds *have been studied in other photosynthetic organisms. In sunflower, the steady-state level of *Psy *was negatively affected by phytoene accumulation [6]. Inhibition of carotenoids synthesis in tomato seedlings resulted in increased *Psy *and *Pds *transcript levels [10], but this up-regulation was not observed in pepper leaves [11].

Carotenoids are essential pigments present in all photosynthetic organisms and protect cells from oxidative damage caused by reactive oxygen species [12]. Nanoparticles cause oxidative damage to macromolecules by mainly increase in ROS production [13]. Therefore, antioxidant defense mechanisms in plants are up-regulated to scavenge ROS and reduce oxidative damage caused by environmental stresses [14].

In the present study, the effects of uncoated and gum-Arabic coated lead sulfide nanoparticles (GA-coated PbS NPs) and light intensity on the mRNA levels of *Pds *in *D. salina *at late logarithmic phase of growth are analyzed by quantitative real time PCR (qRT-PCR) and results are compared with changes in mRNA levels in other photosynthetic organisms.

## MATERIALS AND METHODS

**Algal strain and exposure experiments:** *Dunaliella salina *Teod. strain MSI-3 (GeneBank asccession no. KC477401) which previously was isolated, purified and identified in our laboratory, was cultured in nutrient medium as described by Ben-Amotz et al. [15]. The 250 mL Erlenmeyer flasks, each containing 100 mL of the culture with a cell density of 106 cells ml-1 were exposed to continuous illumination with an intensity of 100 µmol photon m-2 s-1 at 22±2 °C. At late logarithmic phase of growth, uncoated and GA- coated PbS NPs were added to the culture to give 24 µg mL-1 final concentrations. The flasks were immediately transferred to 180 µmol photon m-2 s-1 light intensity and samples were taken at 3, 6, 12 and 48 hrs time intervals for mRNA analysis by qRT-PCR. The size of the PbS NPs and their coating procedures are as described previously [16].

**Total RNA extraction and cDNA synthesis: **Total RNA was extracted from 107 *D. salina *cells using DENA ZIST Asia Kit according to the protocol provided by the manufacturer. To remove genomic DNA,the extracted RNA was treated with DNase (DNase I- RNase free, Fermentas).Using Nano-Drop ND 1000 spectrophotometer (Wilmington, USA), total RNA was quantified at 260 nm and its quality was determined by the A206/A280 ratio and also by gel electrophoresis. Synthesis of cDNA was performed with the First Strand cDNA Synthesis Kit (Fermentas).

**Quantitative real time PCR: **Relative qRT-PCR was carried out with a Line GeneK thermal cycler (Bioer, China). The reaction mixture in a total volume of 20 µl contained 5 µl cDNA, 10 µl 2x GreenStar TM q-PCR Master Mix (Bioneer) and 10 pmole of each primer. The primers for Pds and internal control (18s rRNA) genes were designed using the sequences obtained in GeneBank and Allele ID 7 software. The sequences of primers for these genes are presented in Table 1. For PCR reaction the following thermal profile was used: 94 °C for 10 min, 40 cycles at 94 °C for 10 s, 60 °C for 25 s and 72 °C for 30 s. Specificity of PCR products were confirmed based on melting curves obtained by heating the amplicons from 50 °C to 90 °C. Relative expression of *Pds *was calculated using the equation 2-∆∆Ct in which ∆Ct was obtained by subtracting the internal control Ct value from the Ct value of *Pds.*

**Table 1:** Nucleotide sequences of primers and size of the products in real time PCR amplification

**Genes**	**Primer sequences (5'3')**	**Amplicon length (bp) **
*Pds *F	CTATGACCGTTGTGCTAA	128
*Pds *R	CCTGGAAGTGAAGTAGTT	
18S rDNA F	AGTGTTGGGCAAGTGGAC	148
18S rDNA R	TAGAAATAGCGAGCGAGCG	

**Statistical analyses: **Changes in Pds gene expression under different growth conditions are presented as fold-change relative to control. The experiments were carried out in triplicate and the results are presented as mean ± standard error (SE). Duncan's multiple range tests was used to compare the expression levels. SPSS 16.0 was employed for statistical analyses and p<0.05 was considered as statistically significant.

## RESULTS

**Figure 1:**
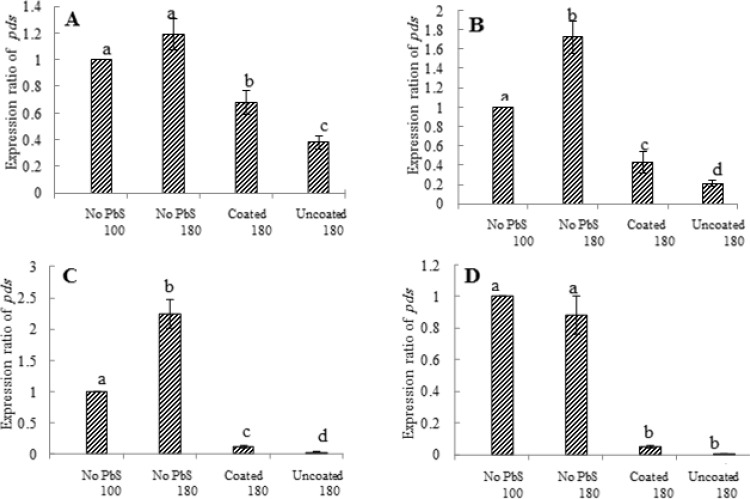
Effects of uncoated and gum-Arabic coated lead sulfide nanoparticles (GA –coated PbS NPs) and light intensity on mRNA levels of phytoene desaturase (*Pds*) in *Dunaliella salina *Teod. Cells grown at 100 µmol photons m-2 s-1 light intensity with no PbS NPs (control culture) were transferred to 180 µmol m-2 s-1 intensity and exposed to PbS NPs. Samples were taken at 3 h (Box A), 6 h (Box B), 12 h (Box C) and 48 h (Box D) after shift to higher light intensity for mRNA analysis

Figure 1 shows changes in the expression levels of the gene encoding Pds in *D. salina *after the algal cells grown at 100 µmol photon m-2 s-1 light intensity (control culture) were transferred to 180 µmol photon m-2 s-1 intensity and simultaneously exposed to uncoated and GA-coated PbS NPs. In the absence of PbS NPs, after 3 h of exposure to higher light intensity, the mRNA level of *Pds *increased by 1.2 fold compared to the control culture, but the increase was not statistically significant at p<0.05. In the presence of the uncoated and GA-coated PbS NPs, significant decrease in the mRNA levels of *Pds *was observed. Exposure to uncoated PbS NPs had more inhibitory effect on the expression level of *Pds* than GA-coated NPs. The expression ratios significantly reduced to about 0.6 and 0.3 in the presence of coated and uncoated NPs, respectively (BOX A).

After 6 h of exposure to higher light intensity, in the absence of NPs, the expression level of *Pds *significantly augmented to 1.7. In the presence of NPs, greater reduction in *Pds *expression was observed when compared to 3 h of exposure; the expression ratios decrease to about 0.4 and 0.2 for coated and uncoated NPs, respectively (BOX B). *D. salina *culture exposed to 180 µmol photon m-2 s-1 light intensity for 12 h, up-regulated *Pds *expression by 2.2 fold compared to cells grown at 100 µmol photon m-2 s-1 intensity (control culture). Continued reduction in mRNA levels in the presence of NPs is noticeable (BOX C). Finally, in the absence of PbS NPs, after 48 h of exposing algal culture to higher light intensity, the mRNA level of *Pds *reduced to that in control culture. Continued reduction in the expression levels of the target gene was observed in the presence of NPs with uncoated PbS NPs essentially abolished *Pds *expression (BOX D).

## DISCUSSION

Phytoene desaturase (Pds) and phytoene synthase (Psy) play important role in the regulation of carotenoids biosynthetic pathway in many photosynthetic organisms [17, 18]. In this study, *Pds *transcript level continued to increase with time reaching a maximum of 2.2-fold, 12 h after shift to higher light intensity. Since *D. salina *was sampled at late logarithmic phase of growth, in addition to higher light intensity, nutrient depletion may have contributed to increase in *Pds *mRNA levels. Increase in *pds *transcript levels due to high light intensity and under nutrient limitation is reported by Coesel et al. in *D. salina *[9]. About 4.5-fold increase in *Pds *mRNA level was observed 48 hr after shift to high light intensity. Combined effects of high light intensity and nutrient limitation caused 8-fold increase in *pds *mRNA after 24 hr of exposure. In addition to *Pds, *high light intensity and nutrient deficiency caused up-regulation of *Psy.*

Contrary findings have been reported in *D.salina *var. *bardavil *[19]. No significant increase in *Pds *mRNA levels or protein level was observed upon exposure of culture to high light intensity. As pointed out by Coesel et al. [9], nutrient levels of algal culture at the time of sampling for mRNA analysis may be responsible for the observed differences in *Pds *response to high light intensity. The up-regulation of carotenoids biosynthetic genes have been demonstrated in other photosynthetic organism. In the Cyanobacterium *Synechococcus *sp. *Psy *and *Pds *were shown to be under transcriptional control. The promoter activity at the *Pds/Psy *operon was higher under strong light [20]. In the unicellular green algae *C. reinhardtii Psy *and *Pds *showed a fast up-regulation in response to light [21]. In the green algae *Haematococous pluvialis*, increase in the Pds protein level, was accompanied by increase in *Pds *mRNA level during the accumulation of ketocarotenoids [22]. It was concluded that *Pds *is regulated at the mRNA level, most likely by transcriptional control.

In accordance with the large production and use of nanomaterials, the number of publications addressing their potential hazards to living organisms and to the environment, especially aquatic environment, has increased rapidly [23]. Generation of ROS has been considered as a general toxicity mechanism for several types of NPs [24, 25]. Coating NPs with substnances like gum-Arabic reduces the toxicity of NPs to organisms [16]. In this work, reduction in *Pds *expression occurred in the presence of PbS NPs. Uncoated NPs had more inhibitory effects on *Pds *mRNA levels compared to the coated NPs. In general, anti-oxidative genes are up-regulated in the presence of NPs [26, 27]. In *A. thaliana*, genomic responses to TiO2 and CeO2 NPs were up-regulation of genes involved in oxidative stress. CeO2 NPs also resulted in down-regulation rather than up- regulation of several genes associated with photosynthesis [14]. Since *Pds *seems to be under transcriptional control in the green algae *D. salina, *genetic manipulation of this gene may result in higher biotechnological production of carotenoids.
